# Akirin2 is essential for the formation of the cerebral cortex

**DOI:** 10.1186/s13064-016-0076-8

**Published:** 2016-11-21

**Authors:** Peter J. Bosch, Leah C. Fuller, Carolyn M. Sleeth, Joshua A. Weiner

**Affiliations:** 1 Department of Biology, The University of Iowa, Iowa City, IA USA; 2 Department of Biology and Department of Psychiatry, The University of Iowa, 143 Biology Building, Iowa City, IA 52242 USA

**Keywords:** Cortical development, Microcephaly, Dorsal telencephalon, Apoptosis, Neuronal differentiation, Neural progenitor

## Abstract

**Background:**

The proper spatial and temporal regulation of dorsal telencephalic progenitor behavior is a prerequisite for the formation of the highly-organized, six-layered cerebral cortex. Premature differentiation of cells, disruption of cell cycle timing, excessive apoptosis, and/or incorrect neuronal migration signals can have devastating effects, resulting in a number of neurodevelopmental disorders involving microcephaly and/or lissencephaly. Though genes encoding many key players in cortical development have been identified, our understanding remains incomplete. We show that the gene encoding Akirin2, a small nuclear protein, is expressed in the embryonic telencephalon. Converging evidence indicates that Akirin2 acts as a bridge between transcription factors (including Twist and NF-κB proteins) and the BAF (SWI/SNF) chromatin remodeling machinery to regulate patterns of gene expression. Constitutive knockout of *Akirin2* is early embryonic lethal in mice, while restricted loss in B cells led to disrupted proliferation and cell survival.

**Methods:**

We generated cortex-restricted *Akirin2* knockouts by crossing mice harboring a floxed *Akirin2* allele with the *Emx1-Cre* transgenic line and assessed the resulting embryos using in situ hybridization, EdU labeling, and immunohistochemistry.

**Results:**

The vast majority of *Akirin2* mutants do not survive past birth, and exhibit extreme microcephaly, with little dorsal telencephalic tissue and no recognizable cortex. This is primarily due to massive cell death of early cortical progenitors, which begins at embryonic day (E)10, shortly after *Emx1-Cre* is active. Immunostaining and cell cycle analysis using EdU labeling indicate that *Akirin2*-null progenitors fail to proliferate normally, produce fewer neurons, and undergo extensive apoptosis. All of the neurons that are generated in *Akirin2* mutants also undergo apoptosis by E12. In situ hybridization for Wnt3a and Wnt-responsive genes suggest defective formation and/or function of the cortical hem in *Akirin2* null mice. Furthermore, the apical ventricular surface becomes disrupted, and Sox2-positive progenitors are found to “spill” into the lateral ventricle.

**Conclusions:**

Our data demonstrate a previously-unsuspected role for Akirin2 in early cortical development and, given its known nuclear roles, suggest that it may act to regulate gene expression patterns critical for early progenitor cell behavior and cortical neuron production.

**Electronic supplementary material:**

The online version of this article (doi:10.1186/s13064-016-0076-8) contains supplementary material, which is available to authorized users.

## Background

The cerebral cortex is a uniquely mammalian structure that is the site of consciousness and higher cognitive functions such as language, memory, and perception. Expansion of the cortex is a hallmark of human evolution: it makes up ~80% of human brain mass, and this increased size is due to alterations in the number, type, and temporal regulation of cortical progenitor cells [1]. Disruptions in the precisely coordinated pattern of progenitor cell behavior during cortical neurogenesis can result in microcephaly and lissencephaly, and are associated with intellectual disability and epilepsy [2]. Perturbation of gene expression patterns in progenitors and nascent neurons during a critical developmental window can have major effects on cortical development, through premature differentiation due to disruption of cell cycle timing [3], altered apoptosis [4] and incorrect migratory signals for newly-born neurons [5]. The recent concern over Zika virus infection, which causes microcephaly by disrupting the proliferation and survival of cortical progenitors [6, 7], highlights the importance of identifying novel molecular mechanisms regulating progenitor behavior.

Akirins are an understudied family of small (22–27 kDa), highly conserved nuclear proteins with demonstrated roles in myogenesis, meiosis, immune function, and gene regulation in *Drosophila*, *C. elegans*, and mammals [8–10]. There are two *Akirin* genes in mammals, *Akirin1* and *Akirin2* [11]; *Akirin1* has also been reported as *Mighty* [12] in mice and *Akirin2* as *FBI1* in rats [13]. Mice harboring a global knockout of the *Akirin1* gene are viable and fertile with no obvious abnormalities; however, global knockout of *Akirin2* results in early embryonic lethality [11]. Though Akirins have a highly-conserved nuclear localization signal, they have no known DNA-binding motifs and appear to regulate gene expression indirectly [10, 14]. In *Drosophila,* Akirin interacts with the transcription factor Twist to control the expression of genes important for myogenesis [8]. Akirins regulate innate immunity in both *Drosophila* [11, 15] and mice (*Akirin2* but not *Akirin1* [11, 16]), by collaborating with NF-κB proteins to control gene expression. Akirin2 was also shown to bind to 14-3-3 proteins, regulators of many intracellular signaling pathways, and to act as a transcriptional co-repressor in this context [13]. Akirin was first reported to act as a bridge between transcription factors such as *Twist* and NF-κB proteins and the SWI/SNF (BAP/BAF) chromatin remodeling complex in *Drosophila* [8]. This was subsequently shown to be conserved in mammals: Tartey et al. found that mouse Akirin2 acts as a bridging protein between the NF-κB and BAF complexes, through an interaction between IκBζ and BAF60 [16]. Akirin2’s role in linking transcription factors and BAF chromatin remodeling machinery is critical for both innate and humoral immune responses in mice, via regulation of gene expression and B cell proliferation and survival [16, 17]. Interestingly, Akirin2 has also been implicated as an oncogene. *Akirin2* is overexpressed in a number of tumor cell lines, and antisense-mediated knockdown of *Akirin2* led to growth inhibition and reduced tumorigenicity and metastasis of K2 hepatoma and Lewis lung carcinoma cell lines [13, 18]. *Akirin2* knockdown also renders glioblastoma cell lines more prone to cell death, suggesting that Akirin2 is important for cell survival in rapidly dividing cells [19].

Though expression databases and tissue northern blots [13] indicate that *Akirin2* is expressed in the brain, Akirins remain entirely unstudied in the nervous system of any organism. The proposed functions of Akirin2 make it particularly interesting as a candidate regulator of cortical development, where progenitor populations divide rapidly in a highly regulated manner and where overlapping patterns of gene expression govern differentiation [20]. It has recently become clear that the mammalian BAF chromatin remodeling complex is a critical regulator of neuronal development. Loss of its core helicase Brg1 in neural progenitors results in an extreme reduction in cortex size [21]. Progenitor proliferation requires the presence of the BAF53A subunit in the BAF complex; a switch from BAF53A to BAF53B is critical for the generation and differentiation of neurons and the elaboration of dendritic arbors by forebrain neurons [22]. Both NF-κB [23] and 14-3-3 proteins [24] have also been reported to regulate the onset and progression of neuronal differentiation in the cortex. Therefore, as a protein that interacts with NF-κB proteins, 14-3-3 proteins, and the BAF chromatin remodeling complex, Akirin2 is well-placed to have an important, previously-undocumented role in cortical development.

Here, we show that Akirin2 is expressed in the embryonic and postnatal cortex, and utilize the *Emx1-Cre* line [25] to conditionally delete the *Akirin2* ^*flox*^ allele [11] in telencephalic progenitors. In the absence of *Akirin2,* mice exhibit extreme microcephaly, with nearly complete absence of any cortical tissue, due to disrupted cell proliferation, reduced neuron production, and massive apoptosis of both neurons and progenitors. Defects appear as early as embryonic day (E)10, soon after the *Emx1-Cre* transgene becomes active [25, 26], and by E12 the only remaining dorsal tissue is a thin epithelium. In situ *hybridization* and immunostaining for markers suggests that the cortical hem may be defective in *Akirin2* knockouts, which could contribute to the phenotypes observed. Additionally, the apical ventricular surface is disrupted, and Sox2-positive mutant progenitor cells are found spilling into the lateral ventricle. This is associated with reductions in connexin-43 and N-cadherin, proteins known to be important for the integrity of progenitor cell-cell contacts at the ventricular surface. Together, our results demonstrate an essential role for Akirin2 in controlling progenitor proliferation, cell survival, and neuron production during cortical development, and suggest that further studies aimed at identifying Akirin2-regulated gene expression patterns will be informative.

## Methods

### Mouse strains

*Akirin2* ^*flox*^ conditional mutant mice [11] were the kind gift of Dr. Osamu Takeuchi, Kyoto University. These were crossed to the *Emx1-Cre* transgenic line [25], obtained from The Jackson Laboratory (JAX stock #005628). *Emx1-Cre* is active in the cortical ventricular zone from ~ E9.5 onwards, and extensive recombination can be seen by E10.5 [26]; this line excises floxed alleles from neural progenitor cells that give rise to all primary glutamatergic neurons and astrocytes in cortex (but not ganglionic eminence-derived GABAergic cortical interneurons). These mice are designated hereafter as *Emx1-Cre; Akirin2* ^*fl/fl*^ in the manuscript and Akirin2 KO (for brevity) in the figures. To label *Akirin2* knockout cells, we often included the *Ai14*-*tdTomato* reporter allele (JAX stock #007914), in which a floxed stop cassette precedes the tdTomato gene inserted into the constitutive Rosa locus. All lines were on a C57BL/6 background. All animal experiments were performed in accordance with the University of Iowa’s Institutional Animal Care and Use Committee and NIH guidelines.

### RT-PCR

Cortical tissue was dissected from mice at the following ages: E11, E12, E15, P0, P10, P28 and Adult. Tissue was placed into TRIzol (Thermo Fisher Scientific) and RNA extracted following manufacturer’s protocols. RNA cleanup was performed using the QIAGEN RNeasy Mini kit according to manufacturer’s protocols. RNA was converted to cDNA using the High Capacity cDNA reverse transcription kit (Applied Biosystems) and PCR was performed using primers in *Akirin2* exons designed to cross multiple intron-exon boundaries to prevent background from any genomic DNA contamination. Primer sequences: Akirin2 Exon 1 to Exon 2, F 5’-CGC CTC GCC GCA GAA GTA TC-3’, R 5’-CAA CCT GGA TCT GCC TGC TGA AA-3’; Akirin2 Exon1/2 junction to Exon 5, F 5’-GCA TCA CCA GGG ACT TCA TCT-3’, R 5’-ACA AAG AAC AAG GCA GCC CA-3’. PCR cycling parameters for 30 cycles were: 95^°^C 1 min, 55^°^C 15 s, 72^°^C 1 min. Quantitative PCR for *Emx1* was performed using cDNA from E15 control and knockout forebrain tissue, Taqman Universal Master Mix (Applied Biosystems) and a validated Taqman primer/probeset (Mm01182609_m1, ThermoFisher) in a Roche Light Cycler 480 following manufacturers instructions. Resulting Emx1 mRNA levels were normalized to levels of β-actin (Mm00607939_s1, ThermoFisher) and analysis of differential expression was performed using the delta delta Ct method to give a fold-change difference in Emx1 expression between control and knockout. Experiments were performed in triplicate and data were analyzed across three separate qPCR experiments.

### Tissue collection and processing

Embryos were collected at E10, E11, E12, E13, E15, E18/P0 and, in a few cases, postnatal ages. Tissues were dissected, immersion fixed with 4% paraformaldehyde (PFA) for 24 h (or perfusion fixed in the case of postnatal animals), washed with PBS and cryoprotected with 30% sucrose. Samples were frozen in OCT and 18–20 μm cryosections were cut on a Leica CM1850 cryostat. Sections were used for in situ hybridization, immunohistochemistry, cresyl violet, or hematoxylin and eosin (H&E) staining.

### In situ hybridization

In situ hybridization was performed with antisense riboprobes using previously published methods [27, 28]. Plasmids containing probes for Ngn2, Lhx2, Lef1, Dmrt3, Wnt3a and TTR were a kind gift of Dr. Elizabeth Grove, University of Chicago. Plasmids containing probes for FGF8 and Wnt5a were a kind gift of Dr. Bernd Fritzsch, The University of Iowa. Probe insert for Akirin2 was generated by RT-PCR of brain cDNA using the following primers: Akirin2: F 5’-CCA ACT ATG ACA TGC AGC-3’; R 5’-GTA CTG TAG ACT AAC TGC-3’. Inserts were cloned into pCR-BluntII-TOPO (Invitrogen) for transcription of sense and antisense riboprobes using digoxigenin-UTP (Roche) per manufacturer’s protocols. Cryosections were post-fixed with 4% PFA, washed and incubated in Proteinase K solution (1 μg/mL, 37^°^C). A second fixation step was performed, prior to washes in PBS and pre-hybridization for 1 h at 70^°^C in hybridization solution (50% formamide, 5× SSC, 1% SDS, 500 μg/mL tRNA, 200 μg/mL acetylated BSA, 50 μg/mL heparin). Overnight hybridization was performed at 70^°^C with the relevant digoxigenin-UTP labeled riboprobe. The following day, sections were washed for several hours at 70^°^C (2× SSC [pH 4.5], 50% formamide, 1% SDS), washed at room temperature (RT) in TBST, blocked with 10% sheep serum for 1 h and incubated at RT for 2 h with anti-digoxigenin-AP antibody (1:5000 in 1% sheep serum, Roche). Sections were washed with TBST, incubated in alkaline buffer (100 mM NaCl, 100 mM Tris-HCl (pH 9.5), 50 mM MgCl_2_, 1% Tween20) for 10 min and then developed using NBT (nitro blue tetrazolium) and BCIP (5-bromo-4-chloro-3-indolyl phosphate) at RT until sufficient color had developed.

### Histological staining

For H&E staining, E12 embryos were fixed in 4% PFA for 3 days, washed with PBS and embedded in paraffin. Samples were sectioned at 7 μm using a Reichert-Jung 2030 Biocut Microtome. Sections were dried onto slides and stained using hematoxylin followed by counterstaining with eosin, dehydration through graded ethanols, and mounting in Permount. For cresyl violet staining, P16 mice were perfusion fixed and brains postfixed with 4% PFA for 24 h, washed with PBS, cryoprotected with 30% sucrose and sectioned at 30 μm using a cryostat. Sections were stained using 0.1% cresyl violet solution, dehydrated through graded ethanols, and mounted in Permount.

### EdU injections

Pregnant dams were injected intraperitoneally with the nucleotide analog EdU (5-ethynyl-2’-deoxyuridine; Invitrogen) at a concentration of 100 μg/g body weight, 12 h prior to embryo collection. Injections were performed when pregnant dams were E10.0 (early morning on E10) and E10.5 (early evening on E10). EdU labeling was detected using the Click-iT® EdU Alexa Fluor® 488 imaging kit (Molecular Probes/Invitrogen), following manufacturer’s instructions.

### Immunofluorescence

Cryostat sections were incubated with blocking buffer (2.5% BSA, 0.01% Triton-X100) for 1 h at RT and incubated with primary antibody diluted in blocking buffer overnight at 4^°^C. Sections were washed with PBS and incubated with secondary antibody for 2 h at RT, washed with PBS and mounted using Fluoro-Gel (Electron Microscopy Services #17985-11). Antibodies used were: Abcam: CTIP2 (ab18465) 1:300; BD Transduction Labs: N-cadherin (610920) 1:500; Cell Signaling Technology: Cleaved caspase-3 (#9661) 1:200, Connexin-43 (#3512) 1:300, Ki67 (#9129) 1:400, phosH3 (#9706S) 1:300; Chemicon: Sox2 (AB5603) 1:400, MAP2 (MAB3418) 1:400, TBR2 (AB9618) 1:400; Covance: Tuj1 (MMS-435P) 1:400, Pax6 (PRB-278P) 1:400. The Pax6 mAb (1:200) developed by A. Kawakami was obtained from the Developmental Studies Hybridoma Bank, created by the NICHD of the NIH and maintained at The University of Iowa, Department of Biology, Iowa City, IA 52242. Sections were incubated in the relevant secondary antibody conjugated to Alexa-Fluor 488 nm, 568 nm or 647 nm (Molecular Probes/Invitrogen) and counter-stained with DAPI (4’,6-diamidino-2-phenylindole) and/or SYTOX® green nucleic acid stain (Molecular Probes/Invitrogen), prior to mounting with Fluoro-Gel.

### Imaging

Confocal and epifluorescence imaging was conducted using a Leica SPE TCS Confocal Microscope and Leica Application Suite software. In situ hybridization and whole mount imaging was conducted using a Zeiss SteREO Discovery V12 microscope and captured using AxioVision Rel4.8 or a Leica DMIRB inverted microscope. Images were adjusted for brightness and contrast using Image/J-FIJI [29, 30] or Adobe Photoshop.

### Quantitative analysis

EdU and Ki67 quantification was performed similar to that described in [31]. Briefly, a region of interest was drawn in the medial portion of the dorsal cortex. Images were thresholded using Image/J and cells counted individually for EdU and/or Ki67 positivity. The cell count was expressed as Ki67 + EdU+/EdU+(%) to assess the percentage of cells that continue to proliferate following EdU incorporation. A total of 2 animals per genotype were used, with 3 sections per genotype.

For quantification of CC3 and Pax6, images were thresholded using Image/J and cells counted within a region of interest in the medial dorsal cortex. DAPI-stained cells were assessed for CC3 or Pax6 to give the number of CC3-positive or Pax6-positive as a percentage of DAPI. A minimum of 2 animals per genotype were used, with a minimum of 3 sections per genotype.

For phospho-histone H3 (PH3) quantification along the apical ventricular zone (VZ) surface, all PH3 positive cells were counted in a 20× confocal image and expressed as a measurement of cells/100 μm. A minimum of two animals per genotype were used, with at least 4 sections per genotype.

Tuj1 cell quantification counted all Tuj1-positive cells in a 10× confocal image and expressed this count as a measurement of cells/100 μm. Two animals per genotype were used, with at least 6 sections per genotype. The Tuj1 line scan analysis was performed in Image/J by drawing 5 lines from the pial surface to the apical VZ surface in a 20× image of each section and using the Plot Profile analysis tool in Image/J to plot intensity along the line. The resulting information was transformed to express intensity of Tuj1 staining across the distance of the total telencephalon (measured thickness of cortical wall (%)). Three animals per genotype were used, with a minimum of three sections per genotype.

For all quantification, unpaired t-tests (Prism software, GraphPad) were used to compare between control and knockout, with significance level *p* < 0.05.

## Results

### *Akirin2* is expressed throughout mouse cortical development

**Fig. 1:**
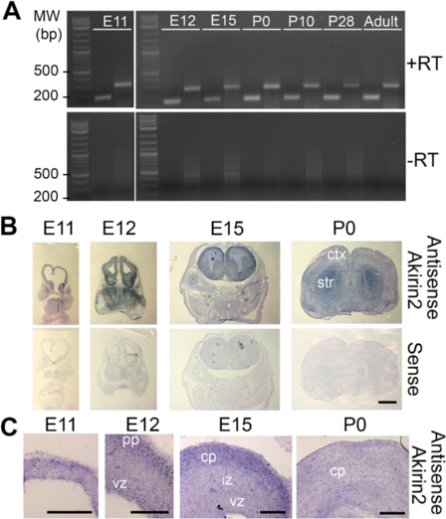
The Akirin2 gene is expressed throughout cortical development. **a** RT-PCR using two different primer-sets that generate amplicons across multiple exons. Akirin2 is expressed in telencephalic/cortical tissue at E11, E12, E15, P0, P10, P28 and in the adult mouse. The -RT control confirms that none of the bands are due to genomic DNA contamination. **b**, **c** Low magnification (**b**) and higher magnification (**c**) images of in situ hybridization experiments utilizing an antisense riboprobe for *Akirin2* indicate that it is expressed throughout the developing telencephalon, with high expression in the early ventricular zone (vz) and in post-mitotic neurons situated in the preplate (pp; E12), intermediate zone (iz) or cortical plate (cp). No appreciable signal was observed using an *Akirin2* sense probe in these or any other experiments. ctx, cortex; str, striatum. Scale bar: 1 mm in (**b**), 200 μm in (**c**)

A previous study presented northern blot data indicating that *Akirin2* is expressed in the adult rat cerebrum and cerebellum [13]. To determine the expression patterns of *Akirin2* in the mouse cortex, we first designed primers to detect *Akirin2* transcripts and performed RT-PCR on telencephalon/cerebral cortex tissue throughout development. Robust expression of *Akirin2* was detected at all ages tested: E11, E12, E15, postnatal day (P)0, P10, P28 and Adult (Fig. 1A). We next performed in situ hybridization to determine the localization of *Akirin2* mRNA in the developing cortex at E11, E12, E15, and P0. *Akirin2* was expressed throughout the cortical wall at all of these ages (Fig. 1B), with particularly strong expression in the early ventricular zone (VZ) and the embryonic preplate/cortical plate, indicating expression by both neural progenitors and postmitotic neurons (Fig. 1C).

### Severe microcephaly in cortically-restricted *Akirin2* knockout mice

**Fig. 2:**
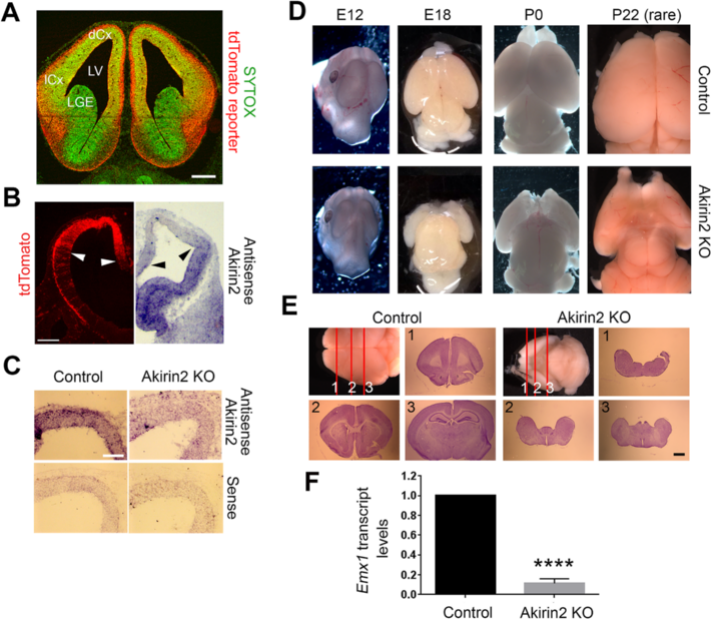
Disruption of the *Akirin2* gene results in loss of the cerebral cortex. **a** A coronal section of the telencephalon of an *Emx1-Cre; Ai14-tdTomato* E12 embryo confirms the restriction of floxed allele excision to the dorsal (dCx) and lateral (lCx) cortex; Sytox Green is used as a cellular counterstain. **b**, **c** Low (**b**) and higher (**c**) magnification views of in situ hybridization utilizing *Akirin2* probes on E11 telencephalon. Loss of *Akirin2* begins in the dorsal cortex (arrows mark similar positions on two different E11 sections showing tdTomato Cre reporter fluorescence and antisense *Akirin2* staining); some *Akirin2* expression is still detected in the lateral cortex at this stage, but is not observed later as Cre activity expands (**b**). In knockout cortex at E11, *Akirin2* antisense signal is reduced compared to controls, and is nearing background sense probe levels (**c**). **d** *Akirin2* knockout causes severe microcephaly, which is already apparent by E12 and which leads to little, if any, remaining cortex in perinatal animals and in the rare animals that survive past P0 (one such knockout that survived to P22 is shown). **e** Cresyl violet staining of a rare surviving Akirin2 knockout and its littermate control at P16. There is no identifiable cortex or hippocampus in the knockout brain, though ventral structures appear to be present (note, for example, the prominent mammillary bodies in the lower right image). **f** Quantitative RT-PCR of control and *Akirin2* knockout E15 forebrain RNA using a Taqman probe set for *Emx1*. Transcripts are nearly absent in the knockout tissue, consistent with loss of the cortex. ****p < 0.0001. LGE, lateral ganglionic eminence; LV, lateral ventricle. Scale bar: 200 μm in (**a**); 200 μm in (**b**); 100 μm in (**c**); 1 mm in (**e**)


To study the role of Akirin2 in cortical development, we crossed an *Akirin2* conditional mutant allele [11] with *Emx1-Cre* [25], which is active in dorsal telencephalic VZ progenitors from ~ E9.5 and has previously been reported to cause substantial gene recombination by E10.5 [26]. Additionally, we included the *Ai14-tdTomato* reporter allele, which expresses this red fluorescent protein from the ubiquitous Rosa locus following Cre excision of a floxed STOP cassette [32], in order to visualize the fate of *Akirin2* knockout cells. As expected, this reporter confirmed the cortex-restricted activity of the *Emx1-Cre* line (Fig 2A, B), and in situ hybridization confirmed substantial reduction of *Akirin2* transcripts within 1–2 days of Cre activation (Fig. 2B, C; note that there are, unfortunately, no validated Akirin2 antibodies appropriate for immunostaining). *Emx1-Cre; Akirin2* ^*fl/fl*^ mice were born in Mendelian ratios but, strikingly, exhibited a near-complete absence of the cortex and did not usually survive past P0 (Fig. 2D). The very few knockouts that survived past birth (we have recovered and examined only four such mice, at P10, P16, P22, and P30) were much smaller than their wild-type littermates, possessed a hunched posture and unsteady gait, and were hyperphagic. The size and behavior of a surviving knockout at P30 is shown in the additional movie file (see Additional file 1: Akirin2 knockout movie). *Emx1-Cre; Akirin2* ^*fl/+*^ heterozygous mice were normal in size and exhibited normal cortical development.

Additional file 1: Akirin2_knockout_movie (MPEG-4). Decreased size, ataxia, and hyperphagia in the few Akirin2 knockouts that survive postnatally. The video shows a P30 Emx1-Cre; Akirin2^fl/fl^ knockout along with control littermates in the home cage. The mutant exhibits a hunched posture, an unsteady gait, and is hyperphagic; we observed that postnatal mutants eat nearly continually. (MP4 12759 kb)


*Akirin2* mutants exhibited a severe loss of dorsal telencephalic tissue, beginning early in corticogenesis. At E12, the reduction of dorsomedial telencephalic tissue was already grossly apparent (Fig. 2D). By E18 or P0 (when most knockout mice die), there was a near-complete loss of the dorsomedial cortex, with only a small amount of remaining ventrolateral tissue of potential cortical origin (Fig. 2D). Gross observation (Fig. 2D; P22) and Nissl staining of sections (Fig. 2E; P16) of the few surviving postnatal *Emx1-Cre; Akirin2* ^*fl/fl*^ knockouts identified no discernible dorsal cortex or hippocampus (Fig. 2E), which are both regions derived from Emx1-expressing cells in the telencephalic VZ [25]. That little, if any, remaining tissue in the knockouts was cortical in origin is confirmed by the almost complete loss of *Emx1* transcripts as assayed by quantitative RT-PCR analysis of E15 forebrain RNA (Fig. 2F). Other brain regions, which did not express Cre and thus remained wildtype for *Akirin2*, appeared grossly normal in size and histological organization (Fig. 2D, E). Some olfactory bulb tissue, which includes many neurons that derive from the cortical VZ, was present in the surviving postnatal animals (Fig. 2D, P22), but the bulbs were greatly reduced in size.

**Fig. 3:**
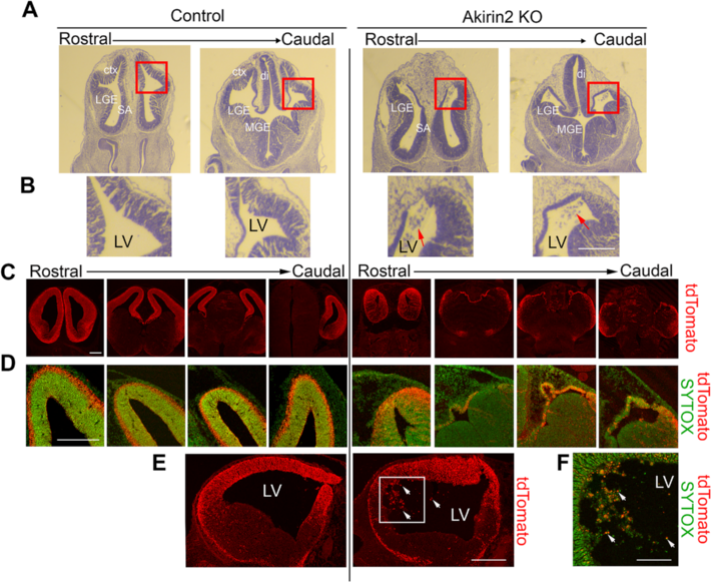
Severe loss of cortical tissue in *Akirin2* knockout embryos. **a** Hematoxylin and eosin staining of coronal sections of control and *Akirin2* knockout cortex (ctx) at E12, shown at two locations on the rostro-caudal axis. The knockout contains only a thin epithelium dorsomedially and exhibits cells falling/spilling into the lateral ventricle (LV). **b** Closeup images of boxed areas in (**a**); *arrows* show cells spilling into the LV. **c**, **d** Comparative low (**c**) and higher (**d**) magnification images of tdTomato reporter and Sytox Green fluorescence in coronal sections of control and *Akirin2* knockout cortex at 4 locations on the rostro-caudal axis. In the knockout, there are still tdTomato cells present, although these are drastically fewer in number and found only laterally, or situated in a thin epithelium dorsally. **e** Control and knockout sagittal sections at E11 showing tdTomato-positive cells. In the knockout at this timepoint, cells are already observed spilling in the LV (*arrows*) and cell loss is more pronounced rostrally than caudally. **f** Higher magnification of boxed area in (**e**); *arrows* show individual cells that are double positive for tdTomato and Sytox Green within the LV. di, diencephalon; LGE, lateral ganglionic eminence; MGE, medial ganglionic eminence; SA, septal area. Scale bar: 200 μm in (**b**, **c**, **d**, **e**); 90 μm in (**f**)

Closer inspection of the histological organization of knockout telencephalon at E12 clearly shows a complete loss of dorsomedial cortex, substantial reduction of ventrolateral cortex, and no apparent effect on the (Cre-negative, and thus *Akirin2* wildtype) lateral or medial ganglionic eminence (LGE/MGE) (Fig. 3A, B). Interestingly, in *Emx1-Cre; Akirin2* ^*fl/fl*^ knockout mice clusters of cells appeared to be “falling into” the lateral ventricle (Fig. 3B, E, F, arrows), indicating disruption of the ventricular surface. All that remained of the mutant dorsal cortex was a thickened epithelium, similar to that previously observed in mice lacking the transcription factor Lhx2 [33]. We next used *Ai14-tdTomato* reporter fluorescence to compare cortical VZ-derived cells between controls and *Akirin2* knockouts. The knockouts contained fewer tdTomato-positive cells at E12, and the largest proportion of these were ventrolateral, due to the already-substantial loss of dorsomedial tissue (Fig. 3C). High magnification images of the knockout telencephalon showed that the thickened dorsal epithelium that spanned the region normally occupied by the growing cortex contained tdTomato-positive cells (Fig. 3D). More tdTomato-positive cells remained ventrolaterally in the E12 knockouts, though there were still very few compared to controls (Fig. 3D). Comparison of sagittal sections of controls and knockouts at E11 (Fig. 3E, F) with the coronal series at E12 (Fig. 3D) suggests that the loss of cells and disruption of the ventricular surface exhibited a rostral-to-caudal temporal gradient; this may, however, simply reflect the time course of *Emx1-Cre* transgene activation, and of subsequent turnover of existing Akirin2 transcripts and proteins.

### Reduced proliferation and increased apoptosis in the developing cerebral cortex of *Akirin2* mutants

**Fig. 4:**
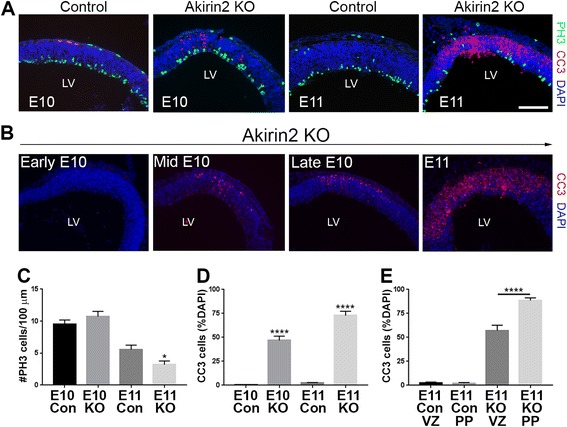
Apoptosis of *Akirin2* knockout cortical progenitors and neurons. **a** Sections from mid-E10 and E11 (**a**) control and knockout cortex were stained for the mitotic marker phospho-histone H3 (PH3; *green*), apoptotic cell marker CC3 (*red*), and DAPI (*blue*). At mid-E10, controls and knockouts exhibit a similar number of PH3+ profiles at the ventricular surface (**c**); a significantly higher number of CC3+ dying cells are observed close to the emerging preplate (**a**, **d**). The cell death progresses between E10 and E11 (**b**), with no apoptotic cells apparent at early E10; CC3+ cells appear at mid-E10, and their number increases at late E10 and peaks at E11. At E11, *Akirin2* knockouts exhibit significantly fewer PH3-positive mitotic cells (**a**, **c**), CC3 staining is greatly increased (**d**, **e**), and the tissue is already thinner (**a**). Further analysis of the CC3+ cells indicates that a greater percentage of cell death occurs in cells close to the pial surface at the developing preplate (PP) compared with cells closer to the apical venticular surface (VZ; **e**). *p < 0.05,****p < 0.0001. LV, lateral ventricle. Scale bar: 200 μm

Because this cortical phenotype was already apparent by E11/12, when cortical neurons are just beginning to be generated, it likely involved disruption of progenitor cell behavior. To determine whether reduced progenitor proliferation and/or increased apoptosis contributes to the observed phenotype of *Emx1-Cre; Akirin2* ^*fl/fl*^ mutants, we stained sections at E10 and E11 for the mitotic marker phospho-histone H3 (PH3) and the apoptotic marker cleaved caspase-3 (CC3; Fig. 4). The *Akirin2* knockout tissue appeared fairly normal at (mid) E10, and no significant difference in the number of PH3+ cells was observed compared to controls (Fig. 4A, C). By E11, however, the number of PH3+ cells was significantly reduced in *Akirin2* knockouts (Fig. 4A, C). While very few CC3+ cells were observed in controls between E10 and E11, staining steadily became more widespread in *Akirin2* knockouts during this 24 h period (Fig. 4A, B, D, E). Examining the time-course of apoptosis in mutant cortex showed, interestingly, that CC3+ profiles appeared first near the pial surface in the developing preplate (mid-to-late E10) before eventually spreading throughout the cortical wall at E11 (Fig. 4B). Even at E11, CC3+ profiles comprise a greater proportion of cells in the preplate (~90% of cells) compared with cells nearer the apical VZ surface (~60% of cells; Fig. 4E). These results suggest that progressive loss of Akirin2 after the activation of the *Emx1-Cre* transgene quickly results in the apoptosis of VZ progenitors and of nascent neurons derived from those progenitors.

**Fig. 5:**
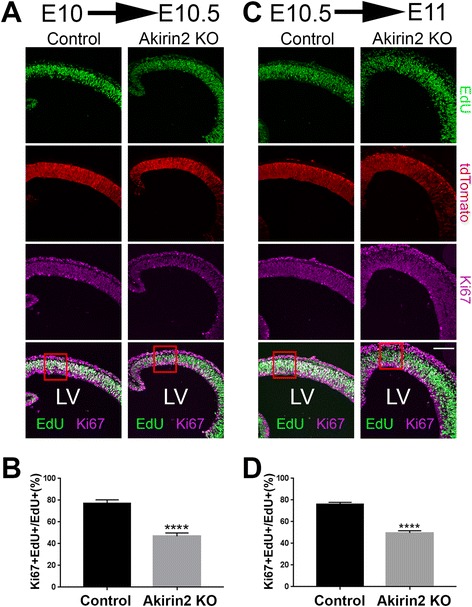
Cell cycle analysis of cortical progenitors in *Akirin2* knockout embryos. Pregnant dams were injected with EdU at E10 and embryos collected 12 h later at E10.5 (**a**, **b**), or injected at E10.5 and embryos collected 12 h later at E11 (**c**, **d**). Coronal sections were double stained for EdU and Ki67 and cells from the medial dorsal telencephalon were assessed for expression of these two markers. The ratio of Ki67 + EdU+/EdU+ cells was significantly reduced in *Akirin2* knockout embryos at both E10.5 (**b**) and E11 (**d**). *Red boxes* in (**a** and **c**) demarcate the regions quantified in (**b** and **d**); all analyzed cells were positive for the Cre reporter, tdTomato. ****p < 0.0001. Scale bar: 100 μm

We next asked if progenitor apoptosis observed in *Emx1-Cre; Akirin2* ^*fl/fl*^ mutants was preceded by cell cycle disruptions by injecting pregnant dams with EdU at either E10 or E10.5, collecting embryos 12 h later, and staining for both EdU and the cell proliferation marker, Ki67 (Fig. 5). Cells that remained in the cell cycle following EdU incorporation would be Ki67+/EdU+, whereas those that exit the cell cycle shortly after EdU incorporation would be Ki67-/EdU+. For both time series, we found a similar significant reduction in the percentage of EdU+ cells that remained Ki67+ (i.e., were still cycling) in the medial dorsal telencephalon (boxes in Fig. 5A, C) of *Akirin2* knockouts compared with controls (Fig. 5B, D). While this is consistent with aberrantly increased cell-cycle exit of *Akirin2* knockout progenitors, we note that some EdU+ mutant profiles appeared to be pyknotic, especially in the E10.5 to E11 experiments. Thus the possibility that some EdU+ cells may appear Ki67- due to undergoing programmed cell death rather than cell cycle exit cannot be excluded. Despite this caveat, given the similar results observed in the E10 to E10.5 experiments when apoptosis is still relatively low (Fig. 4B), our results are consistent with aberrant proliferation of cortical progenitors in the absence of *Akirin2*.

**Fig. 6:**
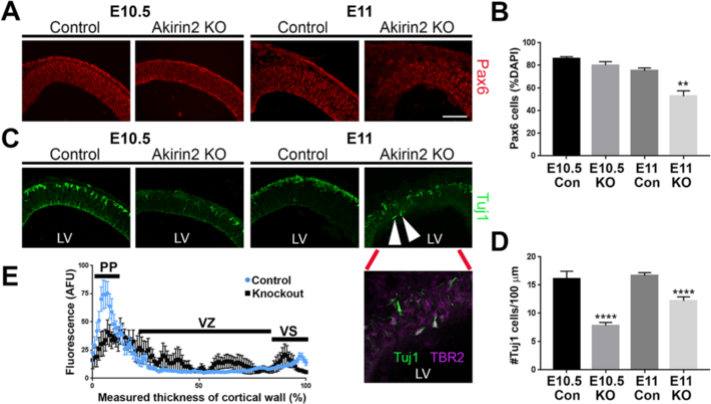
Disrupted production of cortical neurons in *Akirin2* knockout embryos. **a**, **b** The number of Pax6+ progenitors in the *Akirin2* knockout is comparable to controls at E10.5, but significantly reduced by E11 (**a**, **b**). **c**, **d** In contrast, there are already significantly fewer Tuj1+ neurons in knockouts at E10.5; this difference is maintained at E11. Unlike in controls, where all neurons migrate superficially to form a tight preplate (PP) band, at E11 some knockout neurons that double-stain for Tuj1 and TBR2 appear “trapped” at the apical ventricular surface (*arrowheads*, and inset, from **c**). **e** Neurons that do successfully migrate to the PP in the Akirin2 knockout form a less tightly-defined layer (*black lines*) compared with control (*blue lines*), indicated by a line-scan of Tuj1 intensity across the thickness of the cortical wall (**e**). Subsequently, Tuj1 fluorescence intensity is spread more broadly at the PP and is also more apparent at the apical ventricular surface (VS) and throughout the ventricular zone (VZ) in the *Akirin2* knockout. **p < 0.01, ****p < 0.0001. LV, lateral ventricle. Scale bar: 100 μm in (**a**, **c**); 50 μm in (**c**) inset

Staining with CC3 suggested that apoptosis began in the preplate and then spread to the VZ (Fig. 4). To ask whether nascent neurons are lost prior to progenitors, we immunostained control and *Emx1-Cre; Akirin2* ^*fl/fl*^ telencephalon for the radial glial marker Pax6 and the pan-neuronal marker, Tuj1 (antibody recognizing neuronal βIII tubulin). We found that the number of Pax6+ progenitors was similar between controls and knockouts at E10.5 (Fig. 6A, B). Interestingly, the Tuj1+ cell population was already significantly reduced in *Emx1-Cre; Akirin2* ^*fl/fl*^ telencephalon at this stage, suggesting that newly born neurons lacking *Akirin2* die either during or shortly after migration to the emerging preplate (Fig. 6C, D). By E11, both Pax6+ progenitors and TuJ1+ neurons were significantly reduced in knockouts (Fig. 6A–D). Thus, these immunostaining data are consistent with the spread of CC3+ cells observed previously (Fig. 4). At E11 we also noted that *Akirin2* mutants aberrantly exhibited Tuj1+/TBR2+ cells at the apical VZ surface (Fig. 6C, arrows and inset). The Tuj1+ cells that had migrated to the preplate were also not organized in the tight band of cells observed in control tissues. To quantify this, we performed a line scan analysis to measure Tuj1+ staining intensity across the thickness of the telencephalon wall (Fig. 6E). This analysis confirmed that the smaller number of TuJ1+ cells observed in *Akirin2* knockout telencephalon were ectopically placed compared to controls. This could indicate, additionally, aberrant migration and/or differentiation of nascent neurons in the absence of *Akirin2*.

### Disruption of Wnt pathway gene expression in *Akirin2* mutant telencephalon

**Fig. 7:**
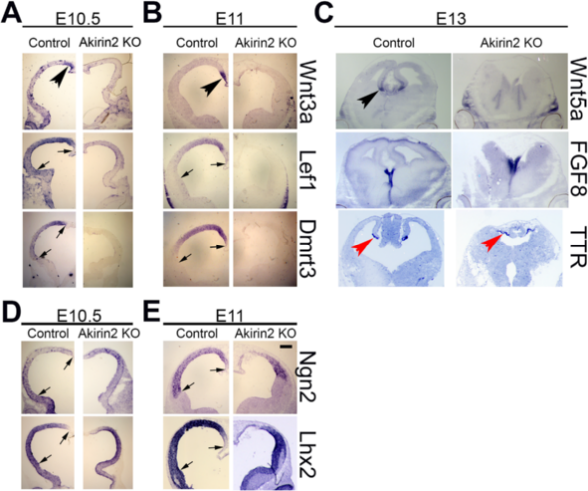
Disruption of the cortical hem in the absence of *Akirin2*. In situ hybridization for a number of cortical patterning genes at E10.5 (**a**, **d**), E11 (**b**, **e**) and E13 (**c**) is shown. The cortical hem, a medial structure that releases Wnt signaling molecules to pattern the cortex, is disrupted in *Akirin2* knockouts, as few if any *Wnt3a* or *Wnt5a* transcripts are detected (**a**, **b**). Consistent with this, the Wnt-responsive genes *Lef1* and *Dmrt3* are reduced or absent in the knockout cortex (**a**, **b**). The *transthyretin* (TTR)-positive choroid plexus, and the ventromedial domain of *Fgf8* expression, both of which are wholly or partially spared by Emx1-Cre, remain present in the knockout (**c**), though the location of the choroid plexus is shifted dorsally due to the loss of cortical tissue. Expression patterns of the proneural transcription factors Ngn2 and Lhx2 appear fairly normal in the dorsal cortical region, although the loss of tissue documented previously is observed at E11 (**d**, **e**). *Arrows* represent the region of Emx1-Cre activity at E10.5 and E11, identified by tdTomato reporter boundaries in other sections not shown. *Black arrowheads* show the Wnt3a and Wnt5a-positive cortical hem and *red arrowheads* show the TTR-positive choroid plexus. Scale bar: 200 μm in (**a**, **b**, **d**, **e**); 300 μm in (**c**)

Cortical size and patterning depends upon Wnts secreted by the medially-situated cortical hem [34]. We thus asked whether the hem (in which the *Emx1-Cre* transgene is active; [25, 34]) might be disrupted in the absence of *Akirin2*. In situ hybridization suggested that this, indeed, was the case, as strong expression of hem markers *Wnt3a* (E10.5 and E11, Fig. 7A, B) and *Wnt5a* (E13, Fig. 7C) was lost. Consistent with this, we also found that the expression of dorsal cortex-enriched Wnt-responsive genes *Lef1* and *Dmrt3* was nearly absent at both E10.5 and E11 in *Emx1-Cre; Akirin2* ^*fl/fl*^ telencephalon (Fig. 7A, B). In situ hybridization indicated no major disruption of the expression pattern of the genes encoding the proneural transcription factor Ngn2 [35] and the LIM-homeodomain transcription factor, Lhx2, an important dorsal telencephalon and cortical patterning gene [33]; the E11 pattern did, however, reflect the cell loss we documented above (Fig. 7D, E). As expected, expression of *FGF8*, a critical cortical patterning gene expressed early in the ventromedial telencephalon [36, 37], and of the choroid plexus marker *transthyretin* (*TTR*) was maintained in *Akirin2* knockout cortex at E13, given that these regions are partially (choroid plexus) or wholly (ventromedial) spared by *Emx1-Cre* [25, 38]. The *TTR*-positive choroid plexus was, however, shifted to a more dorsal and lateral locale due to loss of intervening cortical tissue in *Akirin2* knockouts (Fig. 7C).

### *Akirin2* knockout leads to disruption of the ventricular zone apical surface

**Fig. 8:**
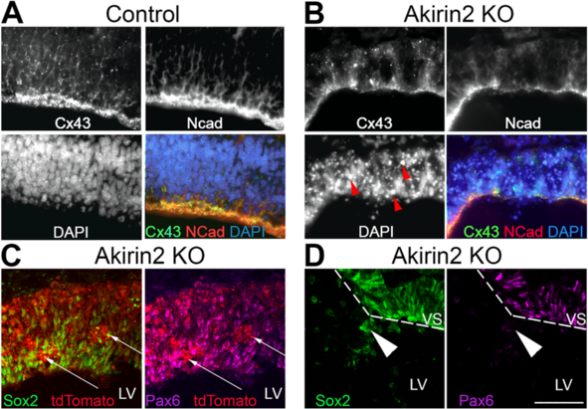
Disruption of the ventricular zone apical surface in *Akirin2* knockout embryos. **a**, **b** High magnification images of the control (**a**) and *Akirin2* knockout (**b**) ventricular zone at E11 stained for N-cadherin and connexin-43. In controls, these proteins are very prominent at apical cell-cell junctions at the ventricular surface, and connexin-43 is also observed at gap junctions through which the cell bodies of progenitors are coupled (**a**). Both proteins are patchy and severely reduced in *Akirin2* knockouts, and are absent at sites where cells are “spilling” into the lateral ventricle (**b**). *Red arrowheads* show chromatin condensation in pyknotic DAPI-stained nuclei observed in the *Akirin2* knockout cortex (**b**). Akirin2 knockout embryos at E11 also display “rosette-like” circular cell clusters within the VZ that contain some Sox2-positive cells but do not stain for Pax6 (*white arrows*; **c**). The cells that spill into the lateral ventricle in *Akirin2* knockout cortex appear to be Sox2+ but Pax6– (**d**, *white arrowheads*). The *dashed line* indicates the apical ventricular surface. LV, lateral ventricle; VS, apical ventricular surface. Scale bar: 50 μm

Finally, we examined further the apparent disruption of the apical surface of the VZ in *Emx1-Cre; Akirin2* ^*fl/fl*^ telencephalon, where cells appeared to spill into the lateral ventricle (Figs 3A, B, E, F and 4A). We immunostained for two proteins, N-cadherin and connexin-43, known to be important for the integrity of the cortical VZ surface. N-cadherin is expressed in neuroepithelial cells, is highly concentrated at their apical adherens junctions, and has an important role in cortical development, as shown by conditional knockout using the forebrain-restricted *D6-Cre* line [39]. N-cadherin loss led to a randomized localization of mitotic and postmitotic cells throughout the cortex, and a very similar disruption of the ventricular surface as that observed in *Akirin2* knockouts [40]. Similarly, the gap-junction protein connexin-43 is important for cortical progenitor cell proliferation, neuronal differentiation, and migration [39, 41, 42]. We suspected that disruption of these genes in *Akirin2* mutants might lead to the observed breakdown of the apical ventricular surface and the spilling of progenitor cells into the lateral ventricle. Indeed, immunostaining revealed disruption of both N-cadherin and connexin-43 at the cortical VZ in *Emx1-Cre; Akirin2* ^*fl/fl*^ mice at E11 (Fig. 8A, B). In controls, there was strong continuous staining of both proteins along the ventricular surface, as well as between radial glia within the VZ. In knockouts, overall levels of N-cadherin and connexin-43 appeared lower and the staining at the ventricular surface was often disrupted at sites where cells were observed spilling into the lateral ventricle (Fig. 8A, B). In addition, we identified circular clusters of cells that appear to have a “rosette-like” morphology within the VZ (Fig. 8C). These rosette-like structures contained some Sox2+ cells, but were not immunoreactive for Pax6. Similarly, the cells within the lateral ventricle near sites of ventricular surface disruption were Sox2+ but Pax6- (Fig. 8D). Together these data suggest that breakdown of the ventricular surface contributes to the phenotype observed in the absence of *Akirin2*, and further suggest that some Sox2+ progenitors proliferate aberrantly as rosettes that may contribute to the disruption of orderly VZ structure required for normal corticogenesis.

## Discussion

Here, we have restricted knockout of the *Akirin2* gene to the developing telencephalon using *Emx1-Cre*, and have demonstrated a critical role for Akirin2 in cortical development. We showed that *Akirin2* is expressed in the mammalian cortex during embryonic and postnatal ages, and that loss of *Akirin2* causes a severe microcephalic phenotype, with complete loss of the dorsomedial cortex and near-complete loss of ventrolateral cortex. Further characterization of the defect shows that it begins early (E10-E11, soon after *Emx1-Cre* becomes active) and leads to impaired progenitor proliferation, reduced neuronal production, and increased apoptosis of both progenitors and neurons. The apical surface of the ventricular zone is disrupted, with cells spilling into the lateral ventricles, and expression of N-cadherin and connexin-43 protein there is patchy and reduced. The vast majority of *Akirin2* knockout mice die at birth, though we recovered a few severely-ataxic mice that survived for up to 4 weeks postnatally.

It is interesting that apoptosis in *Emx1-Cre; Akirin2* ^*fl/fl*^ telencephalon, as measured by CC3 staining and supported by the appearance of pyknotic DAPI profiles, initially occurred near the pial surface at the emerging preplate. In conjunction with reduced Tuj1 staining, it suggests that the earliest-born neurons are lost first in *Akirin2* knockouts, followed hours later by loss of Pax6-positive radial glia progenitors. In addition, the reduced ratio of EdU/Ki67 double-positive cells observed in knockouts suggests an aberrant increase in cells exiting the cell cycle at E10.5-E11. We interpret these results cautiously, as many of the EdU+ profiles analyzed were small and punctate, resembling pyknotic cells and suggesting that some cells may lack significant Ki67 staining due to initiation of apoptosis rather than cell cycle exit per se. Nevertheless, the fact that a significant reduction in cycling cells was observed at E10.5, before apoptosis is massive, is consistent with cell cycle defects in *Akirin2-*null progenitors. Further studies that investigate more closely how *Akirin2* knockout progenitor cells behave in an in vitro environment may allow us better temporal resolution of the transition between cycling progenitors and neurons.

It is interesting to note that *Akirin2* knockout in B cells using *CD19-Cre* leads to reduced Cyclin D1 and Cyclin D2 mRNA expression [17]. Although typically associated with cell cycle progression, Cyclin D1 has also been reported to have a role in promoting neurogenesis in spinal cord that is independent of its cell cycle role [43]. In addition, Cyclin D2 is asymmetrically distributed in daughter cells produced by radial glia cell proliferation in the developing cortex and has a role in G1/S progression. The daughter cell that receives Cyclin D2 maintains its radial glia proliferative state, whereas the other cell undergoes differentiation (reviewed in [44]). Finally, there is evidence that Cyclin D2 is required for the transition from radial glia to intermediate progenitor cells and has a role in proliferation and expansion of the intermediate progenitor pool [45]. Consistent with this, Cyclin D2 knockout mice have a thinner cortex; however, not to the massive extent that we observe with Akirin2 knockout mice [45]. Tightly regulated cell cycle progression is clearly essential in early corticogenesis, as microcephaly can also be caused by disruption of centrosomal proteins [46, 47], cell cycle proteins [48] and mitotic sister chromatid cohesion [49], among others. The rapid loss of telencephalic tissue at a very early age (E10-11) complicates the assessment of whether pro-survival and/or proliferation-promoting genes are specifically mis-regulated in *Emx1-Cre; Akirin2* ^*fl/fl*^ mutants. RNA-seq studies of control and *Akirin2*-null telencephalon at mid-E10 may, however, provide the necessary sensitivity and depth to identify disrupted gene expression patterns leading to cell death.

Based on our current, incomplete, understanding of Akirins in *Drosophila*, *C. elegans*, and mammals, Akirin2 can act as a bridge between a number of transcription factors and chromatin remodeling machinery; therefore, knockout of Akirin2 is likely to affect multiple gene pathways in a cell and tissue-specific manner. Akirins have thus far been linked to Twist [8] and NF-κB [11] transcription factors. Twist is well-studied for its role in cranial development, and loss-of-function Twist1 mutations have been shown to cause Saethre-Chotzen syndrome, characterized by craniosyntosis as well as polydactyly [50, 51]. Interestingly, Twist1 knockout embryos, which die at E11.5, exhibit disruption of the apical neuroepithelial surface and spilling of mitotic cells into the neural tube lumen at E9.5 [52], similar to the disrupted apical VZ surface seen in the *Akirin2* knockout telencephalon. However, there is little evidence from the literature or from our own in situ hybridization data (not shown) that Twist1 is expressed within the telencephalon itself; consistent with this, chimeric embryo studies suggested that neural tube defects in Twist1 knockouts could be rescued cell non-autonomously by introduction of wildtype mesenchymal cells [52]. Still, it is interesting to note that both N-cadherin and connexin-43, which we show are disrupted in *Akirin2* knockouts, are potential Twist1 target genes [53, 54].

NF-κB proteins have well-established roles in the nervous system, being required for synaptogenesis, dendritic spine formation [55] and synaptic function [56]. They also regulate embryonic brain development; interfering with NF-κB signaling results in premature differentiation of cortical progenitors, and subsequent depletion of the progenitor pool [23]. However, it seems unlikely that disruption of NF-κB signaling could be entirely responsible for the observed phenotype of *Akirin2* knockouts: While we see massive loss of dorsal telencephalic tissue between E10 and E12, an NF-κB activity reporter mouse [23] exhibits a pattern that begins ventrolaterally at E11 and even at E13.5 remains patchy dorsally. However, it remains entirely possible that NF-κB interacts with Akirin2 in postmitotic neurons, which also express the *Akirin2* gene (Fig. 1).

In this respect it is interesting to note that in a genome-wide protein interaction screen in *Drosophila*, transactive response DNA-binding protein-43 homolog (TBPH), the homolog of mammalian TDP-43, was identified as an Akirin interactor [57]. Mutations of this protein are associated with amyotrophic lateral sclerosis (ALS) and frontotemporal lobar degeneration [58], and have also been linked to Alzheimer’s disease [59]. TDP-43 is a nuclear DNA and RNA-binding molecule with thousands of RNA targets [60] that is expressed at high levels in the developing cortex and that is required for embryogenesis [61–63]. Postnatal loss of TDP-43 is lethal in mice [64]; in vitro, depletion has been shown to inhibit neurite outgrowth and survival of differentiated N2A cells [65], and to increase the number of dendritic spines in hippocampal neurons [66]. Intriguingly, TDP-43 is highly expressed in the embryonic cortex, but is rapidly downregulated at the end of corticogenesis [67]. It should thus be interesting in future studies to investigate TDP-43 as a potential interactor of mammalian Akirin2 and regulator of corticogenesis.

Perhaps most relevant to the cortical phenotypes we have discovered are the known interactions between Akirins and components of the SWI/SNF chromatin remodeling machinery, referred to as the BAP complex in *Drosophila* and the BAF complex in mammals. BAF is a large, multi-subunit protein complex that modifies chromatin using an ATP-powered core helicase, Brg1. *Drosophila* Akirin interacts with Brahma, the Brg1 homolog, and Twist to regulate gene expression during myogenesis [8]. In mammals, Akirin2 binds to BAF60 subunits, as well as NF-κB protein IκB-ζ, to mediate expression of proinflammatory genes in macrophages [16]. Additionally, in *Akirin2* knockout B cells, Brg1 recruitment to several promoter targets is impaired [17]. The role of Akirin2 in coordinating gene expression via interactions with the BAF chromatin remodeling machinery is likely to be relevant to the cortical phenotypes we have observed here, as a spate of recent studies have uncovered a critical role for the BAF complex in neurogenesis. The neuronal progenitor BAF (npBAF) and neuronal BAF (nBAF) differ in specific subunits that are swapped at the onset of neurogenesis: the npBAF includes BAF53A, BAF45A/D, and SS18, which are replaced in the nBAF by BAF53B, BAF45B/C, and CREST, respectively [68]. This switch in subunits is vital for the transition from progenitor to neuron, as forced expression of BAF53A prevents differentiation of progenitor cells into neurons [21]. In progenitors, the protein repressor-element-1-silencing transcription factor (REST, also known as NSRF) represses neuronal differentiation genes, including the microRNAs miR-9, miR-9* and miR-124 [69, 70], and Brg1 is required for effective REST binding and recruitment to RE-1 sites on target genes [71]. At the onset of neurogenesis, REST and BAF53A are negatively regulated by these three microRNAs and neuronal lineage suppression is lifted [70].

Mutations in a number of BAF subunits genes have been associated with Coffin-Siris Syndrome, a rare autosomal dominant disorder in which microcephaly is observed [72, 73]. Consistent with this, knockout of the Brg1 gene in progenitors (using Nestin-Cre) leads to a smaller cortex; importantly, however, not the complete loss of cortex seen in *Akirin2* mutants [21, 74]. This suggests that the phenotypes we observe following loss of *Akirin2* may reflect both disruption of BAF complex regulation of genes as well as BAF-independent functions of Akirin2. Given the differences in the Tuj1+ and Pax6+ populations in *Emx1-Cre; Akirin2* ^*fl/fl*^ telencephalon at E10.5, it may be that nascent neurons are initially affected to a greater degree than are radial glial progenitors. If Akirin2 is important for the switch to neuron-specific BAF subunits, its loss may lead to apoptosis of neurons that have exited the cell cycle but have not correctly initiated a differentiation program. Clearly, elucidating further the molecular mechanisms through which Akirin2 regulates corticogenesis will require identifying gene expression patterns that are disrupted in its absence. Given the severe and very early loss of most dorsal telencephalic tissue in the *Emx1-Cre-*restricted knockout, this will require developing new conditional knockout lines in which *Akirin2* is disrupted either in smaller numbers of progenitors or in newly postmitotic neurons.

## Conclusions

This study is the first to identify a role for an Akirin in brain development of any organism. We show that the cortex fails to form in the absence of *Akirin2*, due to reduced proliferation and massively increased apoptosis of cortical progenitors accompanied by breakdown of apical ventricular zone structure. Akirin2 may thus be a newly-implicated player in various forms of microcephaly and other malformations of cortical development. Given the known function of Akirins as nuclear proteins that bridge transcription factors and chromatin remodeling machinery, our data suggest that Akirin2 is an important regulator of the gene expression patterns essential for the proliferation and differentiation of cortical progenitors. Our future studies will seek to identify neural genes regulated by Akirin2, identify the proteins with which it interacts in telencephalic cells, and determine the impact of *Akirin2* disruption in postmitotic neuron populations.

## Abbreviations

BCIP: 5-bromo-4-chloro-3-indolyl phosphate
BrdU: 5-bromo-2’-deoxyuridine
CC3: Cleaved caspase-3
DAPI: 4’,6-diamidino-2-phenylindole
EdU: 5-ethynyl-2’-deoxyuridine
LGE: Lateral ganglionic eminence
MGE: Medial ganglionic eminence
nBAF: neuronal BAF
NBT: Nitro blue tetrazolium
NFκB: Nuclear factor kappa-B
npBAF: neuronal progenitor BAF
PFA: Paraformaldehyde
PH3: Phospho-histone H3
PP: Preplate
REST: Repressor-element-1-silencing transcription factor
TBPH: Transactive response DNA-binding protein-43 homolog
TTR: Transthyretin
VS: Apical ventricular surface
VZ: Ventricular zone
